# Editorial: Microbiome and Machine Learning

**DOI:** 10.3389/fmicb.2022.964921

**Published:** 2022-07-05

**Authors:** Isabel Moreno-Indias, Aldert L. Zomer, David Gómez-Cabrero, Marcus J. Claesson

**Affiliations:** ^1^Unidad de Gestión Clínica de Endocrinología y Nutrición, Hospital Clínico Universitario Virgen de la Victoria, Instituto de Investigación Biomédica de Málaga y Plataforma en Nanomedicina-IBIMA Plataforma Bionand, Málaga, Spain; ^2^Centro de Investigación Biomédica en Red de Fisiopatología de la Obesidad y la Nutrición (CIBEROBN), Instituto de Salud Carlos III, Madrid, Spain; ^3^Department of Infectious Diseases and Immunology, Faculty of Veterinary Medicine, Utrecht University, Utrecht, Netherlands; ^4^Navarrabiomed, Complejo Hospitalario de Navarra (CHN), IdiSNA, Universidad Pública de Navarra (UPNA), Pamplona, Spain; ^5^Bioscience Program, Bioengineering Program, Biological and Environmental Science and Engineering Division, King Abdullah University of Science and Technology (KAUST), Jeddah, Saudi Arabia; ^6^School of Microbiology and APC Microbiome Ireland, University College Cork, Cork, Ireland; ^7^SeqBiome Ltd., Cork, Ireland

**Keywords:** microbiome, machine learning, personalized medicine, ML4microbiome, Artificial Intelligence

The human microbiome has attracted more and more attention in the last decade. It has been recognized as a major player in the homeostasis of the host, and in this manner, in the pathophysiology of different diseases. One of the focus points of microbiome research has been advancing the development of personalized medicine approaches, which are potentially necessary to treat multifactorial diseases presenting with heterogenous phenotypes. While significant efforts have been made in terms of sampling, sequencing and analysis multiple well-described disease cohorts and controls, subsequent translation of this information into clinical use has unfortunately been slower than expected.

On the other hand, translation of microbiome insights to clinical practice faces different challenges. For instance, the many different analysis techniques specifically suited for the study of the microbiome have to be standardized, due to the otherwise over-shadowing methodological confounders. A second problem we are facing is that some particularities of microbiome data and its management makes the development of optimized and standardized methods that can deal with this kind of high dimensional data especially difficult. Machine learning (ML) offers great potential to be applied in analyzing these complex datasets. The main goal of the COST Action ML4Microbiome (https://www.cost.eu/actions/CA18131/) is to optimize, standardize and disseminate best practice of ML usage for human microbiome data. This Action has brought together Artificial Intelligence (AI)/ML experts and microbiome researchers to meet this aim, which will ultimately accelerate the advance in the translation of microbiome science.

This endeavor is, however, far from trivial due to several methodological challenges that must first be overcome. Microbiome data are inherently noisy and heterogeneous, there are several different data types, and in most cases many more features (taxa, genes etc.) than samples. In order to describe the current state-of-the-art of ML with microbiome data, Macros-Zambrano et al., reviewed ML use in terms of feature selection, biomarker identification, disease prediction and treatment. The review focused on real ML applications and outlined relevant software and repositories of microbiome data with associated research papers guiding the implementation of future ML efforts in this space. Indeed, ML4microbiome members also expressed their perspective on the past, present and future of the use of ML in microbiome in an accompanying review (Moreno-Indias et al.). Here, the main shortcomings identified were the small size of the datasets used so far, the necessity to combine statistical techniques that have been specifically tailored to fit the particular characteristics of microbiome data, and the need for more user-friendly versions of these approaches to facilitate a wide range usage from different areas of expertise.

Original research manuscripts have also been submitted by the researchers in the field. The contributions published in this Research Topic have both improved current knowledge in particular fields, and contributed with new ML-based tools to be applied in the microbiome space. Some papers focus on the more technical part, including the comparison of Naive Bayes classifiers (NBC) vs. other 16S rRNA taxonomic classifiers based on Random Forest or Neural Networks (Ziemski et al.). The authors demonstrated and concluded that in practical scenarios NBC behave in a similar manner to the other classifiers. Although further improvements will arrive, at least for the moment, NBC use is still guaranteed.

In terms of development of new ML-based tools for enhancing microbiome data analysis have been part of this topic as well, Ramon et al. proposed the *kernInt* package to integrate metagenomic datasets with unsupervised and supervised microbiome analyses, including the recovery of microbial signatures through taxa importance. One important point is that *kernlnt* considers the compositionality of the microbiome data, and that this approach is adaptable enough to use with different applications.

Other applications presented in this Research Topic are two new tools developed based on two disciplines in continuous growth: virome and secretome (Fang and Zhou). Here, the authors used a deep learning approach in order to develop a prokaryote virus virion proteins (PVVPs) prediction tool called VirionFinder, to identify the complete and partial PVVPs from non-prokaryote virus virion proteins (non-PVVPs). The identification of this kind of proteins is a critical step for many viral analyses, such as species classification, phylogenetic analysis and the exploration of how prokaryote virus interact with their hosts. The researchers found that focusing only on a 20 amino acids sequence, instead of the whole or partial proteins VirionFinder, significantly improves sensitivity. Using real virome data further improved the recognition rate of PVVP-like sequences compared to previous tools.

Yu et al. presented their efforts on detecting secreted proteins by Gram-negative bacteria, which is particularly important due to their involvement in bacteria-host interactions. As it is currently challenging to distinguish between different types, especially between type III secreted effectors (T3SEs) and type IV secreted effectors (T4SEs), the authors proposed a deep learning solution for accurately distinguish T3SEs and T4SEs. The tool called DeepT3_4 is able to reach a recall of 80%, providing a promising tool for secretome analysis.

Several manuscripts submitted to this Research Topic have focused on a translational vision. Sudhakar et al. highlighted important computational applications to overcome some of the limitations encountered in microbiome lab-research to enhance our understanding of the microbe-host interactions, and how to fill the big gaps in terms of how the microbiome mechanistically influences host functions at both system and community levels (Sudhakar et al.). This comprehension allows us to progress the development of biomarkers uncovering mechanisms for therapeutic interventions and generating integrated signatures to stratify patients. Other authors have focused on particular diseases, such as Bakir-Gungor et al., who used different supervised and unsupervised ML models to investigate novel microbiota to find Type 2 diabetes (T2D) biomarkers. They increased the diagnostic accuracy and identified several species from *Bacteroides* and other genera that were relevant for the disease. These bacteria have been previously reported to play roles in T2D pathophysiology.

Finally, Vilne et al. presented a minireview on the use of ML in coronary artery disease and its risk prediction. The authors discussed the inclusion of diet-gut microbiome interactions in order to advance development of personalized medicine. Although microbiome data is of paramount importance for the development of a precision medicine approach, they argued that there are still several hurdles to take related to the homogenization of the data, both in terms of microbiome and diet. Once these have been addressed, the development of wearable biosensors for the patients' self-care may be possible.

In conclusion, the introduction of the use of ML in microbiome research is still in its infancy and much more research and methods development are necessary. These new approaches hold great potential for predicting individual health status, the Research Topic presented in this issue will hopefully aid in accelerating the transition. The ML4microbiome COST Action has made great strides in bringing the microbiome and ML community together which can lead to the necessary advancements in both research communities.

## Author Contributions

All authors listed have made a substantial, direct, and intellectual contribution to the work and approved it for publication.

## Funding

This study was supported by the COST Action CA18131 “Statistical and machine learning techniques in human microbiome studies”. IM-I was supported by the “MS type II” program (CPII21/00013) from the Instituto de Salud Carlos III and co-funded by Fondo Europeo de Desarrollo Regional-FEDER.

## Conflict of Interest

MC was employed by company SeqBiome Ltd., Cork, County Cork, Ireland. The remaining authors declare that the research was conducted in the absence of any commercial or financial relationships that could be construed as a potential conflict of interest.

## Publisher's Note

All claims expressed in this article are solely those of the authors and do not necessarily represent those of their affiliated organizations, or those of the publisher, the editors and the reviewers. Any product that may be evaluated in this article, or claim that may be made by its manufacturer, is not guaranteed or endorsed by the publisher.

